# Potential of Sodium-Glucose Cotransporter-2 Inhibitors in the Management of Heart Failure With Preserved Ejection Fraction: A Narrative Review

**DOI:** 10.7759/cureus.73906

**Published:** 2024-11-18

**Authors:** Abdulaziz F Alsuwayh, Mohammed Altawili, Marwan Fahad Alhazmi, Dhuha Faisal M Alotaibi, Alghamdi Omar Rashed, Alharbi Hussam Obaid Abdullah, Ahad Ahmad N Alkenani, Rudayna adel S Almohammdi, Hamad Fahad M Alotaibi, Fatima Essamaldin Altahir Mohamed Alsharif

**Affiliations:** 1 Cardiology, King Salman Armed Forces Hospital, Tabuk, SAU; 2 General Practice, Alroda Primary Health Care Center, Tabuk, SAU; 3 Cardiology, King Abdulaziz Hospital, Jeddah, SAU; 4 General Practice, Ras Tanura General Hospital, Ras Tanura, SAU; 5 Cardiology, Albaha University, Albaha, SAU; 6 General Practice, Riyadh Second Cluster, Al Artawiyah General Hospital, Riyadh, SAU; 7 Cardiology, King Abdulaziz University, Jeddah, SAU; 8 General Practice, Urgent Care Center at Train Station, Al Madinah, SAU; 9 General Practice, University of Jeddah, Jeddah, SAU; 10 General Practice, Buraidah Central Hospital, Buraydah, SAU

**Keywords:** efficacy, heart failure, heart failure with preserved ejection fraction, semaglutide efficacy, sodium-glucose cotransporter-2 (sglt-2) inhibitors

## Abstract

Heart failure with preserved ejection fraction presents a major clinical challenge due to its complex pathophysiology and limitations in its therapeutic options. This comprehensive review explores the comparative effectiveness of sodium-glucose cotransporter-2 inhibitors, focusing on their impact on diabetic versus non-diabetic patients, individuals with chronic kidney disease, and the elderly. A comprehensive literature search identified randomized controlled trials, meta-analyses, and clinical studies that evaluated the role of sodium-glucose cotransporter-2 inhibitors in managing heart failure with preserved ejection fraction. Findings indicate that sodium-glucose cotransporter-2 inhibitors significantly reduce heart failure hospitalizations and cardiovascular mortality. Among chronic kidney disease patients with heart failure with preserved ejection fraction, sodium-glucose cotransporter-2 inhibitors showed a decrease in the risk of cardiovascular mortality and hospitalization. Furthermore, their use in elderly patients was associated with improved health-related quality of life and cognitive function, with no notable increase in adverse events. Clinical guidelines increasingly recommend sodium-glucose cotransporter-2 inhibitors as part of heart failure with preserved ejection fraction management. However, further research is required to refine patient-specific strategies and explore additional benefits, such as their cardioprotective role post-myocardial infarction. This review highlights the effectiveness of sodium-glucose cotransporter-2 inhibitors in managing heart failure with preserved ejection fraction across various subgroups. Additionally, integrating these agents into clinical practice has significant potential to improve patient outcomes.

## Introduction and background

Heart failure (HF) is a widespread disease affecting over 26 million individuals globally [1]. Cardiologists classify HF according to left ventricular ejection fraction (LVEF) into three categories: heart failure with preserved ejection fraction (HFpEF) when LVEF ≥50%, with reduced ejection fraction (HFrEF) when LVEF <40%, and mid-range ejection fraction (HFmrEF) when LVEF is 40-50% [2]. Over 50% of HF patients are diagnosed with preserved ejection fraction, and its frequency is increasing alongside the rising prevalence of metabolic conditions such as obesity, diabetes, and hypertension [3-5]. HFpEF is a clinical syndrome characterized by HF symptoms and signs in the absence of substantial left ventricular systolic dysfunction. Patients with HFpEF frequently experience significant symptoms, including fatigue, dyspnea, and exercise intolerance, which greatly diminish their quality of life (QoL) [6,7]. This condition is associated with frequent hospitalizations, substantial healthcare costs, and increased mortality, further highlighting the need for targeted treatment strategies. Unlike HFrEF, HFpEF lacks well-established therapeutic strategies, making it a significant challenge in cardiovascular (CV) medicine [6,8]. Historically, pharmacological interventions in HFpEF have focused on managing comorbidities such as hypertension and diabetes, with limited success in altering the underlying disease progression [6,8].

Over the past decade, sodium-glucose cotransporter-2 (SGLT2) inhibitors have emerged as a viable treatment for HF, particularly HFpEF. SGLT2 inhibitors were initially developed for managing type 2 diabetes mellitus (T2DM). They have exhibited various cardioprotective effects irrespective of glucose regulation in diabetic patients. SGLT2 inhibitors have a direct impact on the pathophysiological mechanisms of HF. By reducing fluid overload through their diuretic effect, they are mitigating myocardial inflammation and improving vascular function [9,10]. Therefore, SGLT2 inhibitors hold promise for enhancing clinical outcomes in patients with HFpEF.

Recent extensive clinical trials, such as the EMPEROR-Preserved and the DELIVER, have provided compelling evidence for the efficacy of SGLT2 inhibitors in improving CV outcomes in patients with HFpEF, particularly in decreasing hospitalization rates and overall mortality [11,12]. These findings have significantly expanded the therapeutic landscape for HFpEF, which previously lacked targeted treatments. This comprehensive review explores the clinical applications and effectiveness of SGLT2 inhibitors in managing HFpEF. Additionally, we examine the role of these drugs in different populations, such as patients with diabetics, the elderly, and those with chronic kidney disease (CKD), highlighting the evolving landscape of HF treatment.

## Review

Methods

We searched the following databases: PubMed, Web of Science, Scopus, and Embase to identify peer-reviewed articles published between 2014 and 2024 related to the use of SGLT2 inhibitors in HFpEF. The search strategy incorporated the following keywords to ensure comprehensive coverage of the relevant topics. The following terms were used: “Sodium-Glucose Cotransporter-2 Inhibitors,” “SGLT2 Inhibitors,” “SGLT2i,” “Sodium-Glucose Transporter 2 Inhibitors,” “Sodium-Glucose Cotransporter Type 2 Inhibitors,” “Gliflozins,” “Dapagliflozin,” “Heart Failure with Preserved Ejection Fraction,” and “HFpEF.” Then, all authors summarized the recent articles based on their focus on the clinical significance and therapeutic applications of SGLT2 inhibitors in HFpEF.

Pathophysiology of HFpEF

A considerable percentage of the population with chronic HF displays maintained EF, defined by normal LV systolic function. HFpEF constitutes a more complex HF phenotype than HFrEF, mostly linked to diastolic dysfunction [13]. According to observational studies, HFpEF accounts for at least 40-50% of all HF patients, with a higher prevalence in older groups [13-15]. Every 10 years, the incidence and prevalence of HFpEF are predicted to increase by 10% compared to HFrEF [16]. 

The clinical manifestations of HFpEF emerge from complex pathophysiological alterations in the structure and function of cardiomyocytes, resulting in hemodynamic impairment [17]. Nevertheless, the pathophysiology of the disease extends the cardiomyocyte, involving multiple organ systems and contributing to its complexity. The etiology of HFpEF and HFrEF is very different, with HFpEF being a heterogeneous clinical syndrome rarely occurring due to a single identifiable clinical condition [17]. Therefore, it usually develops in patients with various risk factors [17]. Old age has a greater impact on the development of HFpEF than HFrEF [18,19].

Additionally, HFpEF may manifest in individuals younger than 65 years, which might indicate a distinct age-based phenotype [20]. Arterial hypertension is a significant risk factor for the development of HFpEF, affecting individuals across all age groups [21]. Diabetes and obesity are significant risk factors and indicators of HFpEF [22]. Obesity is a disproportionately large risk factor affecting HFpEF. Typically, patients with obesity and HFpEF have younger age profiles [23]. However, the precise pathogenic contribution of each risk factor remains inadequately understood.

When such conditions demand an elevation in cardiac output that exceeds the limitations of the compromised heart, the likelihood of acute pulmonary and systemic venous congestion is significantly evaluated [24]. Despite the great morbidity linked to HFpEF, there have been a few advancements in its management [25]. Instead of using diuretics to reduce congestion and treat co-morbidities, novel pharmacological treatments may soon be available.

Mechanism of action of SGLT2 inhibitors

Nearly all of the body's glucose and most of the sodium filtered by the glomerulus are reabsorbed through SGLT2 transporters. These transporters are only found on the renal proximal convoluted tubules. The blockage of these transporters results in a diuretic action that leads to the excretion of glucose and sodium. This mechanism provides a method for reducing serum glucose levels that does not rely on insulin and does not increase the risk of hypoglycemia [26]. The effectiveness of SGLT2 inhibitors in reducing hemoglobin A1c by 0.5% to 1.1% has been demonstrated by randomized clinical trials (RCTs). In the past two decades, canagliflozin, ertugliflozin, dapagliflozin, and empagliflozin have received approval for use in the management of T2DM. In accordance with the mandates set forth by the U.S. FDA in 2008, SGLT2 inhibitors were obligated to undergo outcome trials to ascertain that these medications did not elevate the risk of CV disease. This requirement stemmed from the discovery that rosiglitazone, a promising thiazolidinedione, increased the risk of CV events [27].

Following a median interval of 3.1 years, 7,020 patients with CV diseases were included in the outcomes studies conducted to assess CV outcomes with empagliflozin. The main composite endpoint of major adverse cardiovascular events (MACE-3) demonstrated a 14% decrease with the administration of empagliflozin. The reduction was primarily due to a 38% decline in CV mortality. Empagliflozin also notably reduced all-cause mortality by 32%. Empagliflozin was also shown to dramatically lower HF hospitalizations (HHF) by 35% [28]. These results were consistent, particularly concerning the decrease in HHF, suggesting that SGLT2 inhibitors may possess a distinct role in the treatment of HF. 

Clinical trials of SGLT2 inhibitors and HFpEF

The Sotagliflozin in Patients With Diabetes and Recent Worsening Heart Failure (SOLOIST-WHF) trial was the first study demonstrating that SGLT2 inhibitors may diminish cardiovascular events in individuals with both HFpEF and T2DM [29]. Following this, the EMPEROR-Preserved trial specifically assessed the efficacy of SGLT2 inhibitors, particularly empagliflozin, in HFmrEF and HFpEF patients, regardless of their diabetes status [12]. This trial demonstrated that empagliflozin significantly reduced the risk of HHF by 27%, resulting in a 19% reduction in the composite endpoint of CV mortality and HHF. The improvement in the primary outcome was apparent as early as 18 days after the initiation of treatment [30]. Nonetheless, the trial observed a reduced advantage in patients exhibiting an LVEF exceeding 60%, indicating that the medication might demonstrate greater efficacy in individuals situated at the lower spectrum of the normal LVEF range [31]. 

The DELIVER trial evaluated the effectiveness of dapagliflozin in patients with HFmrEF and HFpEF. The design largely mirrored that of the EMPEROR-Preserved experiment, with the significant addition of patients with enhanced ejection fraction [32]. Dapagliflozin demonstrated a substantial reduction in the key composite outcome compared to placebo, primarily attributable to a 21% decrease in HHF and urgent care visits. In contrast to the EMPEROR-Preserved trial, the DELIVER study exhibited a consistent benefit in patients with LVEF ≥60%, along with a notable decrease in the main outcome.

Impact of SGLT2 Inhibitors on HFpEF Symptoms and QoL

SGLT2 inhibitors have shown remarkable efficacy in managing symptoms and improving QoL in patients with HFpEF. Depending on pooled analysis, SGLT2 inhibitors were shown to significantly reduce HHF [10,33,34]. The HR for the composite of CV death and HHF was 0.78, indicating a significantly reduced incidence of HHF alone. Moreover, there is a notable reduction in symptoms related to HF. Conversely, improvements in CV mortality and the risk of all-cause death did not achieve statistical significance [34]. The extensive safety profile of SGLT2 inhibitors enhances their use, showing a significantly lower risk of severe adverse events compared to placebo [34]. The results highlight the practical significance of SGLT2 inhibitors in the management of HFpEF, as they effectively alleviate symptom burden, decrease hospitalizations, and enhance patient-reported outcomes. 

Subgroup Analysis of the Recent Clinical Trials 

Subgroup analyses from the EMPEROR-Preserved trial underscored the reliable efficacy of SGLT2 inhibitors across diverse patient populations, encompassing individuals with various renal functions. In individuals exhibiting compromised renal function, the study revealed a notable decrease in adverse CV outcomes [35]. These results emphasize the broad applicability of SGLT2 inhibitors in managing HFpEF across diverse patient subgroups. Additionally, the study’s secondary endpoints, including health-related QoL measures, showed significant improvements, highlighting the positive impact of SGLT2 inhibitors beyond CV outcomes [36]. 

The DELIVER trial similarly confirmed the effectiveness of dapagliflozin in diminishing the risk of CV mortality and HHF, with consistent benefits across age groups and baseline renal function [37,38]. Moreover, dapagliflozin's nephroprotective effects were evident, as it contributed to a deceleration in the deterioration of renal function over time, especially among individuals exhibiting moderate renal impairment. The trial also demonstrated the drug’s efficacy across different glycemic statuses, further supporting its utility in HFpEF management regardless of glucose levels [39]. 

Frailty, a critical factor influencing patient outcomes, was addressed in the DELIVER trial, where frail individuals experienced greater improvements in patient-reported outcomes, particularly in QoL measures such as the Kansas City Cardiomyopathy Questionnaire (KCCQ) [40]. These findings further reinforce dapagliflozin's role as an effective treatment option for diverse patient groups, including frail individuals [37,41].

*Role of Body Mass Index* *in SGLT2 Inhibitors and Semaglutide Efficacy*

There is an apparent contradiction arising from the nuanced interpretation of how body mass index (BMI) affects the response to SGLT2 inhibitors in patients with HFpEF. Subgroup analyses from the EMPEROR-Preserved trial suggest that BMI significantly influences the treatment response. However, the comprehensive findings suggest that empagliflozin consistently demonstrates efficacy in mitigating the risk of HHF and CV mortality, regardless of the patients' BMI [42]. The DELIVER trial also demonstrated that SGLT2 inhibitors had a consistent treatment effect on the primary outcome across different BMI groups, with HR indicating a reduction in risk for both HHF and CV death, regardless of BMI classification [42-44]. Additionally, the findings indicated that patients with a higher BMI might experience more pronounced symptom relief and improvements in QoL metrics, such as KCCQ-CSS, compared to those with lower BMI [43,44].

In contrast, findings from the Semaglutide Trial in People with Obesity and HFpEF (STEP-HFpEF) trial indicate that semaglutide markedly enhanced symptoms, physical limitations, and exercise capacity while concurrently decreasing body weight in individuals with obese HFpEF [45]. The administration of semaglutide led to an average enhancement of 16.6 points in KCCQ-CSS, in contrast to an increase of 8.7 points observed with the placebo. The projected disparity between the two cohorts was 7.8 points (95% CI: 4.8-10.9; p<0.001), signifying a statistically meaningful enhancement in health-related QoL associated with semaglutide therapy [45]. Notable advancements in the six-minute walk distance were observed, with patients receiving semaglutide exhibiting a change of 21.5 m, compared to a mere 1.2 m in the placebo cohort (estimated difference: 20.3 m; p-value <0.001) [45]. The findings indicate that the advantages of semaglutide are strongly dependent on the degree of weight reduction, thereby underscoring its efficacy as a treatment approach for individuals with obese HFpEF [45].

Therefore, BMI influences certain aspects of treatment response and symptom relief, but empagliflozin remains broadly effective across various BMI categories in reducing HHF and CV death, affirming its role as a valuable therapeutic option for HFpEF patients irrespective of their weight status [42,44]. Meanwhile, it presents a promising approach specifically for those with obesity-related HFpEF, demonstrating significant improvements across multiple health metrics (Table 1) [45].

**Table 1:** Comparative analysis of key outcomes and findings from the EMPEROR-Preserved and DELIVER clinical trials on SGLT2 inhibitors in heart failure with preserved and mildly reduced ejection fraction. HFpEF: heart failure with preserved ejection fraction; HFmrEF: heart failure with mid-range ejection fraction; LVEF: left ventricular ejection fraction; CV: cardiovascular; HHF: heart failure hospitalizations; HR: hazard ratio; CI: confidence interval; eGFR: estimated glomerular filtration rate; KCCQ-OSS: Kansas City Cardiomyopathy Questionnaire-Overall Summary Score; QoL: quality of life; SGLT2: sodium-glucose cotransporter-2.

Trial	EMPEROR-Preserved trial	DELIVER trial	Reference
Patient population	HFpEF (LVEF ≥40%)	HFpEF and HFmrEF (LVEF >40%)	[41,46]
Number of patients	5,900+	6,263	[41,46]
Primary outcome	A 21% decrease in the composite outcome of CV events or HHF was observed (HR 0.79, p<0.001).	Decrease in CV mortality or worsening in HF incidents (HR 0.77-0.81 across various subgroups)	[36,37,39]
Hospitalizations	27% reduction in total HHF	Significant reduction in HHF, consistent across age and eGFR categories	[35,37,41,47]
Subgroup findings	Uniform advantages observed among renal function groups: a 17% decrease in adverse CV events (HR 0.83)	Consistent effectiveness observed across various age groups and renal function, particularly in those with eGFR 45-60 mL/min/1.73 m² (HR 0.68)	[38,48]
Renal function	No adverse effects on renal outcomes	Slowed decline in eGFR, with nephroprotective benefits (HR 0.68 for eGFR 45-60 mL/min/1.73 m²)	[38]
Glycemic status	No major focus on glycemic status	Efficacy across normoglycemia, prediabetes, and diabetes (HR 0.77-0.81)	[39]
Patient-reported outcomes	KCCQ-OSS improved by 1.97 points (p<0.001); the six-minute walk test improved by 10.78 m (p=0.032)	KCCQ-OSS improvement is more significant in frail patients (3.4 points at four months, p=0.021)	[49,50]
Frailty subgroup	No specific frailty analysis reported	Greater benefits in frail patients, particularly in QoL measures (KCCQ-OSS)	[50]

Comparative use of SGLT2 inhibitors in special populations

Use of SGLT2 Inhibitors in Diabetic Versus Non-diabetic Patients

SGLT2 inhibitors have emerged as a significant therapeutic option in the treatment of HFpEF, with their efficacy observed across different patient populations, including those with and without diabetes. Recent studies have demonstrated the comparative effectiveness of SGLT2 inhibitors between these groups, highlighting distinct outcomes and clinical implications [51-56]. In diabetic patients, SGLT2 inhibitors have exhibited significant effects on CV outcomes. Clinical trials indicate a 27% decrease in HHF and a 15% decrease in CV mortality, which is a statistically significant reduction [51,52]. The findings highlight the effectiveness of SGLT2 inhibitors in reducing acute HF incidents and improving long-term CV health in patients with T2DM [57]. 

Preliminary findings from the SOTA-P-CARDIA trials are yielding significant insights regarding the impact of SGLT2 inhibitors on non-diabetic patients. The results of this study are enhancing our comprehension of the therapeutic possibilities of SGLT2 inhibitors, extending beyond their recognized application in diabetic groups [58]. This trial focuses on assessing changes in LV mass and other cardiac parameters among non-diabetic individuals to determine if the benefits observed in diabetic patients can also apply to those without diabetes. Initial results indicate that SGLT2 inhibitors significantly improve echocardiographic parameters, such as LV mass and global longitudinal strain, in diabetic patients [58]. Additionally, current data indicate that SGLT2 inhibitors lead to improvements in cardiac function for non-diabetic patients, including reductions in HHF rates similar to those seen in diabetic patients [58,59].

A comprehensive analysis of eight RCTs, encompassing 5,233 non-diabetic patients, revealed a 20% reduction in the relative risk (RR) of CV deaths and HHF among individuals administered SGLT2 inhibitors in comparison to control groups (RR 0.78; p<0.001) [60]. Additionally, SGLT2 inhibitor therapy was linked to significant metabolic benefits, comprising a decrease in body weight of −1.21 kg (P<0.001) and a reduction in systolic blood pressure of −1.90 mm Hg (p=0.04) [60]. However, the agents did not exert a significant influence on CV mortality rates or stroke outcomes in non-diabetic patients, as evidenced by the lack of notable changes in NT-proBNP levels and other CV biomarkers [60,61]. Although there are encouraging findings concerning the application of SGLT2 inhibitors in both groups, additional investigation is essential to better understand their effectiveness and formulate detailed treatment protocols customized for each patient group (Table 2).

**Table 2:** Comparative analysis of SGLT2 inhibitors in diabetic versus non-diabetic patients. CV: cardiovascular; HHF: heart failure hospitalizations; HR: hazard ratio; CI: confidence interval; SOLOIST-WHF: Sotagliflozin in Patients With Diabetes and Recent Worsening Heart Failure; SCORED: Sotagliflozin in Patients With Diabetes and Chronic Kidney Disease.

Parameter	Diabetic patients	Non-diabetic patients	Reference
Reduction in HHF	27% reduction (HR: 0.73; p<0.001)	21% reduction (HR: 0.79; p=0.001)	[51,52,55]
Reduction in CV mortality	15% reduction (HR: 0.85; p=0.001)	18% reduction (HR: 0.82; p=0.005)	[51,53]
Composite endpoint reduction	35% reduction (HR: 0.65; p<0.001)	30% reduction (HR: 0.70; p<0.001)	[51]
Reduction in left ventricular mass	Mean reduction: 6.4 g (95%; p<0.001)	Mean reduction: 5.2 g (95%; p=0.01)	[52,55]
Improvement in diastolic function	Significant improvement observed	Significant improvement in ongoing studies	[52,55,56]
Changes in echocardiographic parameters	Improvement in global longitudinal strain and structural changes	Preliminary findings suggest improved global longitudinal strain	[57]
Impact on systolic function	Improvement noted in systolic function	Data pending; ongoing trials like SOLOIST-WHF and SCORED will clarify	[51,58,62]

Effectiveness in CKD Patients

SGLT2 inhibitors have shown considerable CV and renal advantages in individuals with CKD, including various HF phenotypes, including HFpEF. Extensive data from numerous large-scale clinical trials have consistently underscored the effectiveness of SGLT2 inhibitors in reducing HHF and CV mortality among patients with both CKD and HFpEF [38,63-65]. 

A comprehensive meta-analysis assessing the cardioprotective properties of SGLT2 inhibitors in individuals with CKD stages 3 and 4 indicated a 26% decrease in the risk of CV mortality and HHF, with particularly significant reductions observed in the CKD stage 3a (30%) and stage 3b (23%) subgroups [65]. Additionally, patients with T2DM and CKD experienced a 29% reduction in adverse CV outcomes, while those with HFpEF showed a 28% risk reduction, underscoring the broad therapeutic efficacy of SGLT2 inhibitors across different CKD stages and comorbid conditions [65].

Dapagliflozin demonstrated a notable reduction in the deterioration of renal function, exhibiting a significant difference of 1.4 mL/min/1.73 m² per year when compared to the placebo group, highlighting its renoprotective effects [38]. Thus, SGLT2 inhibitors provide significant CV and renal benefits for CKD patients with HFpEF. The robust reduction in HF-related outcomes and the nephroprotective properties observed in both early and advanced stages of CKD highlight the potential of these agents in managing this high-risk population [38,65,66].

Efficacy of SGLT2 Inhibitors for Elderly Populations

Elderly patients with HFpEF face distinct challenges due to the higher prevalence of comorbidities and frailty. SGLT2 inhibitors have shown consistent benefits across age groups, including those aged 75 years and older. The DELIVER trial indicated that dapagliflozin markedly diminished the risk of CV mortality in individuals with HFpEF without any notable age interaction [37]. Similarly, in the EMPEROR-Preserved trial, empagliflozin demonstrated a RR reduction across age categories, including patients over 80 (HR 0.73) [67]. The efficacy of SGLT2 inhibitors extends to enhancing health-related QoL and functional status among the elderly population. This trial also reported that empagliflozin improved KCCQ scores across all age groups, with particularly early improvements noted in patients aged over 80 years [50,67]. Cognitive and mood improvements have also been observed in elderly patients treated with SGLT2 inhibitors, as demonstrated by significant improvements in Montreal Cognitive Assessment (MoCA), Mini-Mental State Examination (MMSE), and Geriatric Depression Scale (GDS) scores after six months of treatment (p<0.0001) [68]. Safety outcomes were consistent across age groups in both trials, with no significant increase in adverse events such as hypotension, hypoglycemia, or genitourinary infections in elderly patients [37,67]. These findings suggest that SGLT2 inhibitors are effective and well tolerated in elderly HFpEF patients, providing important cardiovascular, functional, and cognitive benefits.

Pharmacological recommendations and clinical guidelines

SGLT2 inhibitors have recently been incorporated into clinical recommendations for the management of HFpEF. Data from numerous RCTs indicate that SGLT2 inhibitors significantly reduce the occurrence of HHF and improve cardiovascular outcomes in patients with HFpEF [10,69]. The results of the EMPEROR-Preserved and DELIVER trials support the implementation of SGLT2 inhibitors as a suggested pharmacological approach for patients with HFpEF, especially for those who present with additional risk factors such as diabetes and hypertension [17,51]. SGLT2 inhibitors are acknowledged for their capacity to enhance diastolic function and alleviate comorbidities linked with HFpEF, including obesity and T2DM [23,55,57]. Despite the promising results, current guidelines still need to clarify the specific indications for SGLT2 inhibitors in HFpEF.

The integration of SGLT2 inhibitors into treatment algorithms for HF management represents a significant advancement in therapeutic strategies. Traditionally, treatment for HF has focused on managing fluid overload and controlling blood pressure through diuretics and antihypertensive agents [16]. However, the introduction of SGLT2 inhibitors offers a novel approach that targets multiple pathophysiological mechanisms associated with HFpEF [9]. Clinical evidence suggests that SGLT2 inhibitors not only reduce hospitalizations but also improve functional status and QoL for HFpEF patients [24,49,63]. Consequently, they may serve as fundamental drugs in conjunction with existing treatments, such as beta-blockers and angiotensin-converting enzyme inhibitors [69]. Thus, while further research is warranted to fully elucidate the long-term benefits of SGLT2 inhibitors in HFpEF populations, current evidence supports their role as a key component in the evolving treatment landscape for HF [4,63,69]. The integration of these agents into clinical practice guidelines reflects a growing recognition of their potential to enhance patient outcomes across diverse populations affected by HF. 

Future directions in HFpEF and SGLT2 inhibitor research

Beyond the effectiveness of SGLT2 inhibitors in HF, their potential applications in CV disease are presently being investigated. Following myocardial infarction (MI), SGLT2 inhibitors may have a cardioprotective effect, according to experimental research. In patients randomly assigned 72 hours post-percutaneous coronary intervention (PCI), empagliflozin in patients with EMMY study exhibited a statistically significant decrease in blood NT-proBNP levels during 26 weeks following empagliflozin administration [70]. The influence of SGLT2 inhibitors on cardiovascular outcomes in post-MI patients is presently being assessed in two ongoing trials: EMPACT-MI and DAPA-MI [71].

The glucagon-like peptide-1 (GLP-1) agonist is a distinct category of glucose-lowering drug that has proven effective in decreasing the risk of CV events in people with T2DM who are at heightened risk. Although this class of drugs does not exert the same influence on heart failure as SGLT2 inhibitors, they possess renoprotective characteristics and facilitate significant weight loss. The American Diabetes Association (ADA) recommends the use of GLP-1 receptor agonists in T2DM patients at elevated risk for CV events, similar to SGLT2 inhibitors. The concurrent administration of SGLT2 inhibitors and GLP-1R agonists is presently under investigation to determine whether combined antagonism of multiple adverse metabolic pathways may yield a synergistic effect and provide additional benefits. Two recent clinical trials have assessed the synergistic effect of dulaglutide and SGLT2 inhibitors in patients with poorly managed type 2 diabetes mellitus. The AWARD-10 trial evaluated dulaglutide as an adjunct to SGLT2 inhibitors [72], whereas the DURATION-8 trial examined the efficacy of weekly exenatide combined with daily dapagliflozin against each medication administered separately in individuals inadequately managed on metformin monotherapy [73]. Both studies demonstrated that co-administration of these drug classes does not elevate the risk of hypoglycemia. Furthermore, they were determined to be beneficial in enhancing glycemic control without a notable rise in adverse events.

## Conclusions

Using SGLT2 inhibitors in managing HFpEF has demonstrated significant effect, showing benefits across a wide range of patient subpopulations. This review highlights their efficacy in reducing HHF, improving CV outcomes, and enhancing QoL for patients with HFpEF. Moreover, the expanding evidence base advocates for incorporating SGLT2 inhibitors into current clinical guidelines for managing HFpEF.

The successful integration of these agents into individualized treatment regimens, alongside the insights gained from ongoing clinical trials, will play a pivotal role in enhancing HFpEF management and optimizing patient outcomes on a wider scale. However, there are some challenges during their use, such as optimizing the use of SGLT2 inhibitors in diverse clinical scenarios and understanding their long-term safety profile. So, further research is needed to refine treatment strategies, focusing on patient selection and appropriate dosing in special populations.
